# *α*-Integrin expression and function modulates presentation of cell surface calreticulin

**DOI:** 10.1038/cddis.2016.176

**Published:** 2016-06-16

**Authors:** C-C Liu, P Leclair, M Monajemi, L M Sly, G S Reid, C J Lim

**Affiliations:** 1 Department of Pediatrics, University of British Columbia, Vancouver, BC, Canada V5Z 4H4; 2 Department of Medicine, University of British Columbia, Vancouver, BC, Canada V5Z 4H4; 3 Michael Cuccione Childhood Cancer Research Program, BC Children's Hospital, Vancouver, BC, Canada V5Z 4H4

## Abstract

Calreticulin presentation on the cell surface is an important hallmark of immunogenic cell death (ICD), serving as the prophagocytic signal for macrophages. Cell adhesion is a physiologically relevant stimulus previously shown to increase calreticulin interaction with *α*-integrins via the juxtamembrane, cytosolic GFFKR motif. This study assessed whether integrin function can regulate surface calreticulin levels in ICD. We generated calreticulin-null T-lymphoblasts and confirmed the loss of surface calreticulin expression on cells treated with doxorubicin, an ICD inducer. Reconstituted expression with full-length calreticulin targeted to the endoplasmic reticulum (ER) successfully rescued doxorubicin-induced surface calreticulin. Reconstitution with a truncation mutant calreticulin targeted to the cytosol led to constitutively high surface calreticulin that was not further elevated by doxorubicin, suggesting calreticulin released from the stressed ER transits the cytosol before its translocation to the cell surface. When stimulated to engage integrin substrates, doxorubicin-treated wild-type T-lymphoblasts exhibited decreased surface calreticulin compared with cells under non-adherent conditions. The inhibitory effect on surface calreticulin was recapitulated for cells in suspension treated with a *β*1-integrin-activating antibody, 9EG7. Similarly, cells expressing a truncated *α*-integrin cytosolic tail, bearing only the juxtamembrane GFFKR calreticulin-binding motif, exhibited low surface calreticulin with doxorubicin treatment under non-adherent conditions. Using partial permeabilization techniques to distinguish between cytosolic and ER staining, we found that ICD inducers promoted the accumulation of cytosolic calreticulin with negligible change in total calreticulin, suggesting that integrin-mediated inhibition of surface calreticulin was due to reduced cytosolic to surface translocation. T-lymphoblasts co-treated with an ICD inducer and 9EG7 exhibited reduced phagocytosis by macrophages when compared with treatment with only ICD inducer. This study reveals a previously uncharacterized function of integrins as negative regulators of ICD by suppressing presentation of cell surface calreticulin.

Immunogenic cell death (ICD), or immunogenic apoptosis, is a form of cell death promoted by anthracycline-based chemotherapy, radiotherapy, and photodynamic therapy.^1, 2^ Unlike non-immunogenic or tolerogenic apoptosis, ICD of tumor cells promotes their engulfment by professional phagocytes, leading to the activation of T cells and subsequent antitumor immune responses.^3^ Such antitumor immune response could be beneficial for the treatment of metastatic diseases, where immune-based recognition of a primary treated tumor leads to targeting of tumors at a secondary site, a phenomenon known as the abscopal effect.^1, 4^ The key difference between non-immunogenic and immunogenic apoptosis is the presentation and release of damage-associated molecular patterns (DAMPs) to the extracellular cell surface and milieu.^5^ DAMPs have various mechanisms of action that include serving as a chemoattractant and the phagocytic ‘eat me' signal for macrophages and dendritic cells to initiate the response to ICD.^6, 7, 8^

Calreticulin (CRT) is a 46 kDa Ca^2+^-binding protein and molecular chaperone that is highly enriched within the lumen of the endoplasmic reticulum (ER).^9^ The N-terminal domain carries a cleavable 17-amino-acid signal peptide that targets its insertion into the ER lumen.^10^ Further enrichment of CRT within the ER is facilitated by a C-terminal KDEL ER-retention motif. The functional attributes of CRT outside of the ER remain a subject of intense interest.^11^ When decorated on the surface of cells undergoing ICD, surface CRT is a DAMP that elicits the phagocytic response.^1, 6, 7^ Compared with the ER, cytosolic CRT is of low abundance,^10^ but its presence is supported by studies highlighting its intracellular transport^10, 12^ and requirements for the C-domain for the cytosolic localization.^13^ More recently, somatic frameshift mutations resulting in CRT with a novel C terminus lacking the KDEL motif were described for a large subset of patients with myeloproliferative neoplasms.^14, 15^

Integrins are *αβ*-heterodimeric transmembrane cell adhesion receptors involved in numerous cellular functions including survival, growth, differentiation and apoptosis.^16^ As such, integrin function has a pivotal role in the development of chemotherapeutic resistance and cancer relapse.^17, 18^ A physiological switch for integrin-mediated signaling is cell adhesion, which is facilitated by the activated, or high-affinity ligand binding, state of the integrins. Activated integrins undergo conformational changes that modulate the binding of effector proteins to the *α*- and *β*-integrin cytosolic tails.^19^ The tails of *α*-integrins share few sequence similarities, with the exception of the conserved membrane-proximal GFFKR motif that forms the inner membrane clasp with its *β*-subunit counterpart.^20^ Several studies, including ours, have shown that integrin-mediated adhesion promotes binding of CRT, likely from the relatively low abundant cytosolic pool, to *α*-integrin tails in a manner requiring the GFFKR motif.^21, 22, 23, 24^ In this study, we investigated if *α*-integrin function may impact upon cell surface presentation of CRT using T-acute lymphoblastic leukemia (T-ALL) cells, which are amenable to cell adhesion studies and cytotoxic drug treatments to induce ICD. We present evidence suggesting that adhesion-mediated integrin–CRT interaction occurring in the cytosol constitute a mechanism reducing CRT translocation to the cell surface, which has implications in antitumor immunomodulatory activity.

## Results

### Doxorubicin induces surface CRT presentation in Jurkat T-lymphoblasts

**Figure 1:**
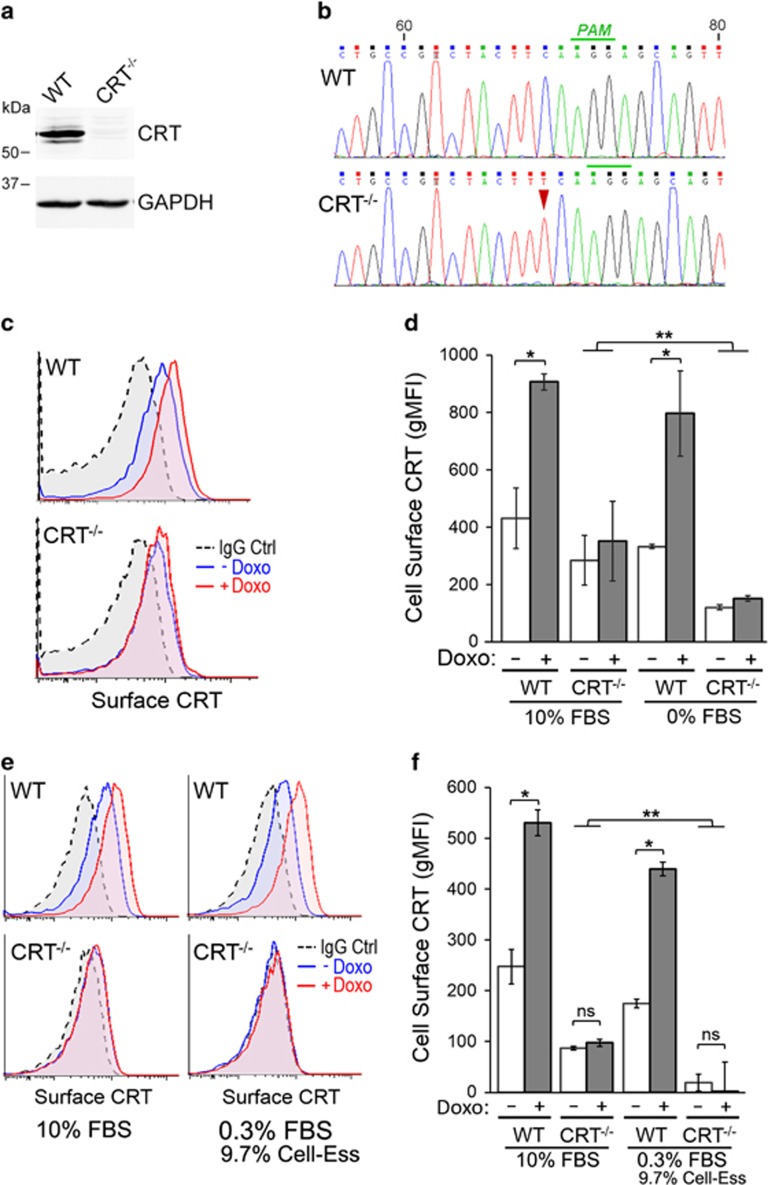
Doxorubicin (Doxo) treatment induces cell surface CRT expression in Jurkat T cells. (**a**) Western blot analysis of lysates from Jurkat WT and CRISPR-Cas9 generated CRT^−/−^ cells. (**b**) Sequencing of *CALR* genomic loci showing single-nucleotide insertion (red triangle) occurring at 68 bp from the predicted start codon and −3 bp from the PAM recognition motif (green bar). The frameshift-mutated variant encodes for a predicted 58-amino-acid protein product because of a premature termination codon. (**c**) Representative flow cytometry plots of surface CRT comparing WT and CRT^−/−^ cells cultured in 10% FBS-supplemented RPMI, untreated or treated with doxorubicin (Doxo). (**d**) Flow cytometry gMFI plots of surface CRT for the indicated cells cultured in either 10% FBS-supplemented RPMI or serum starved for 24 h (0% FBS), and untreated or treated with doxorubicin. Plotted are the mean±S.D.; *n*=3; *P<0.01; **P<0.05. (**e**) Representative flow cytometry plots of surface CRT comparing WT and CRT^−/−^ cells cultured in 10% FBS media or in 0.3%FBS/9.7% Cell-Ess serum replacement media (Essential Pharmaceuticals, Ewing, NJ, USA), untreated or treated with Doxo. (**f**) Flow cytometry gMFI plots of surface CRT as described in (**e**). Plotted are the mean±S.D.; *n*=3; *P<0.001; **P<0.05; NS=not significant. Data shown in (**c**–**f**) are representative of three independently conducted experiments. IgG, immunoglobulin G

To study ICD drug-mediated stimulation of surface CRT presentation in T-ALL cells, we used CRISPR-Cas9-mediated genome editing^25^ to silence CRT expression (CRT null (CRT^−/−^)) in Jurkat T-lymphoblasts. Western blot analysis and sequencing of the *CALR* loci confirmed the loss of CRT expression resulting from a frameshift insertion and a predicted early translation termination (Figures 1a and b). To assess the effects of anthracycline on cell surface CRT, cells were treated with doxorubicin, and surface CRT assayed by flow cytometry on live, non-permeabilized cells. Doxorubicin-treated wild-type (WT) cells exhibited ~2-fold increase, whereas CRT^−/−^ cells exhibited no change in surface CRT levels (Figures 1c and d).

Despite lacking CRT expression, CRT^−/−^ cells exhibited a significant level of surface CRT when compared with the staining control. As this *α*-CRT-reactive signal was present in the serum-supplemented media (Figure 1d and Supplementary S1), we adapted and cultured cells in a serum-free synthetic replacement media. In a final culture state consisting of 0.3% fetal bovine serum (FBS) and 9.7% Cell-Ess, CRT^−/−^ cells no longer exhibited any significant staining for surface CRT, with or without doxorubicin treatment (Figures 1e and f). Doxorubicin-mediated stimulation of surface CRT in WT cells remained equally robust (~2-fold increase) in 10 or 0.3% FBS-cultured conditions, indicating that most, if not all, of the surface CRT stimulated by anthracycline and detected with the *α*-CRT antibody is endogenous to the cell. These results indicate that anthracycline treatment of a T-leukemic cell line increased CRT trafficking to the surface from endogenous stores, and that this phenomenon is abrogated in cells lacking CRT expression.

### Doxorubicin-mediated increase in surface CRT requires the ER-resident form

**Figure 2:**
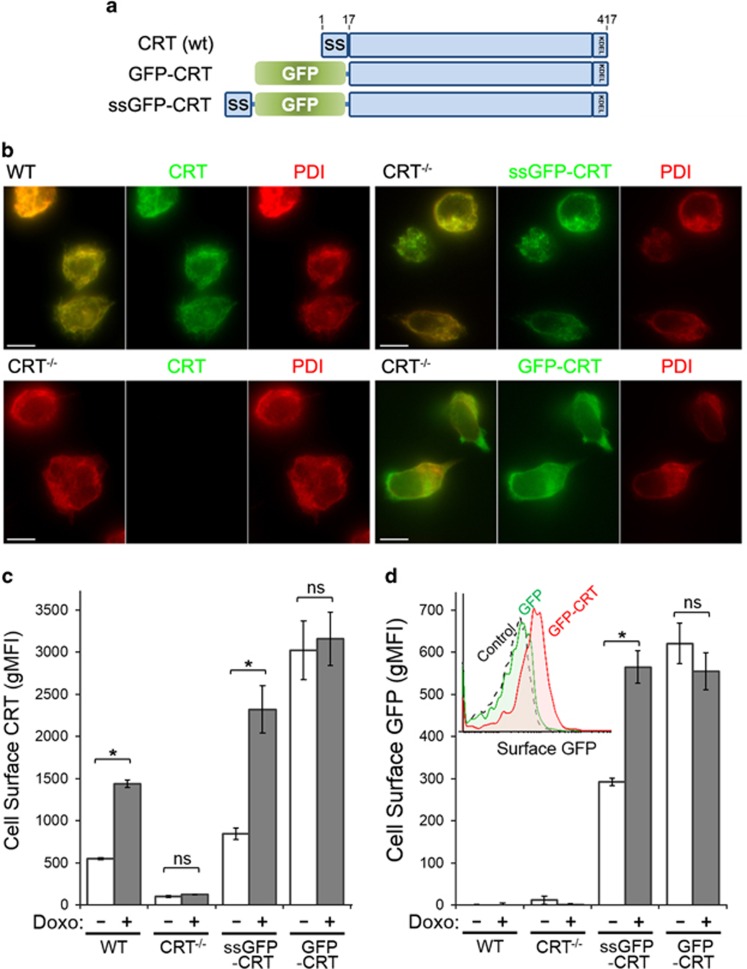
Doxorubicin (Doxo)-mediated increase in surface CRT requires ER-resident CRT. (**a**) Schematic of WT CRT, GFP-CRT (cytosol-targeted) and ssGFP-CRT (ER-targeted). (**b**) The indicated cells were fixed, permeabilized with Triton X-100 and immunostained as follows. Left panels: Immunofluorescence images of endogenous CRT (green) and PDI (red) in Jurkat WT and CRT^−/−^ cells. Right panels: CRT^−/−^ cells transiently transfected to express ssGFP-CRT or GFP-CRT (green) were stained for PDI (red). First column shows color-merged images. Bars: 5 *μ*m. (**c**) Flow cytometry gMFI plots of surface CRT on live WT, CRT^−/−^ and CRT^−/−^ cells expressing ssGFP-CRT or GFP-CRT, untreated or treated with doxorubicin. (**d**) Same as (**c**) but cells were stained with antibodies targeting cell surface GFP instead of CRT. Inset is a representative flow cytometry plot of control non-transfected cells, and cells transfected to express GFP-CRT or GFP alone, which was stained for surface GFP with antibodies. Plotted are the mean±S.D.; *n*=3; *P<0.01; NS=not significant. Data shown in (**b**–**d**) are representative of three independently conducted experiments

The N-terminal 17-amino-acid signal sequence (ss) of CRT is required for synthesis and insertion of the CRT propeptide into the ER lumen, whereas CRT expressed without ss is enriched in the cytosol.^10^ To decipher the source of surface CRT in live cells, we used green fluorescent protein (GFP)-tagged CRT that was targeted either to the ER (ssGFP-CRT) or the cytosol (GFP-CRT) (Figure 2a). To validate their expected localization and targeting, CRT^−/−^ cells transfected to express ssGFP-CRT or GFP-CRT was costained for protein disulfide isomerase (PDI), an ER-resident marker. For comparison, we stained for endogenous CRT and PDI in WT and CRT^−/−^ cells. As expected, PDI exhibited colocalized staining with endogenous CRT in WT cells, and with ssGFP-CRT expressed in CRT^−/−^ cells (Figure 2b). In contrast, PDI colocalized poorly with GFP-CRT in CRT^−/−^ cells (Figure 2b), indicating that ssGFP-CRT targeted to the ER, whereas GFP-CRT remained diffuse in the cytosol.

Next, we analyzed surface CRT levels on live CRT^−/−^ cells reconstituted with the ER-targeted ssGFP-CRT or the cytosolic-targeted GFP-CRT by flow cytometry. Expression of ssGFP-CRT restored the ability for doxorubicin-mediated stimulation of surface CRT in CRT^−/−^ cells at ~2-fold levels, comparable to WT cells (Figure 2c). In contrast, CRT^−/−^ cells expressing GFP-CRT exhibited high surface CRT levels that was not further increased upon doxorubicin treatment (Figure 2c). To confirm the functionality of the GFP-tagged CRT, we used GFP antibodies in place of CRT antibodies to detect the fusion GFP-CRT when presented on the surface of live, non-permeabilized cells. Non-transfected GFP-negative cells showed only background signals, whereas surface staining with GFP antibodies for CRT^−/−^ cells expressing ssGFP-CRT or GFP-CRT were similar to that observed with CRT antibodies (Figures 2c and d), indicating that the fusion proteins were translocated to the cell surface and behave comparably to endogenous CRT. As a further control, we show that GFP was not present on the surface of CRT^−/−^ cells expressing GFP alone (inset in Figure 2d).

These results show that ss-mediated targeting of CRT to the ER facilitated the increased surface CRT that was stimulated by doxorubicin. When targeted directly to the cytosol, high levels of CRT were present at the surface in a manner that was not responsive to doxorubicin. Taken together, the data suggest a mechanism whereby doxorubicin-induced ER stress leads to CRT release from the ER lumen into the cytosol, following which CRT presentation on the surface is directly dependent on its increasing cytosolic concentration.

CRT is thought to be functionally pleiotropic with multiple cellular localizations. We assessed, and found that loss of CRT in T-lymphoblasts did not alter doxorubicin-treated extracellular release of ATP, a DAMP (Supplementary Table 1).^26^ Similarly, loss of CRT and expression of the cytosolic GFP-CRT in T-lymphoblasts did not affect the expression of major histocompatibility complex (MHC) class I antigens (Supplementary S2).^27^ In murine cells, anthracycline-stimulated surface translocation of ER-resident protein 57 (ERp57) is dependent on CRT.^28, 29^ We assessed ICD-induced surface ERp57 and found no significant difference between WT and CRT^−/−^ lymphoblasts, or CRT^−/−^ cells expressing GFP-CRT (Supplementary S3).

### Expression and function of *α*-integrin reduces cell surface CRT

**Figure 3:**
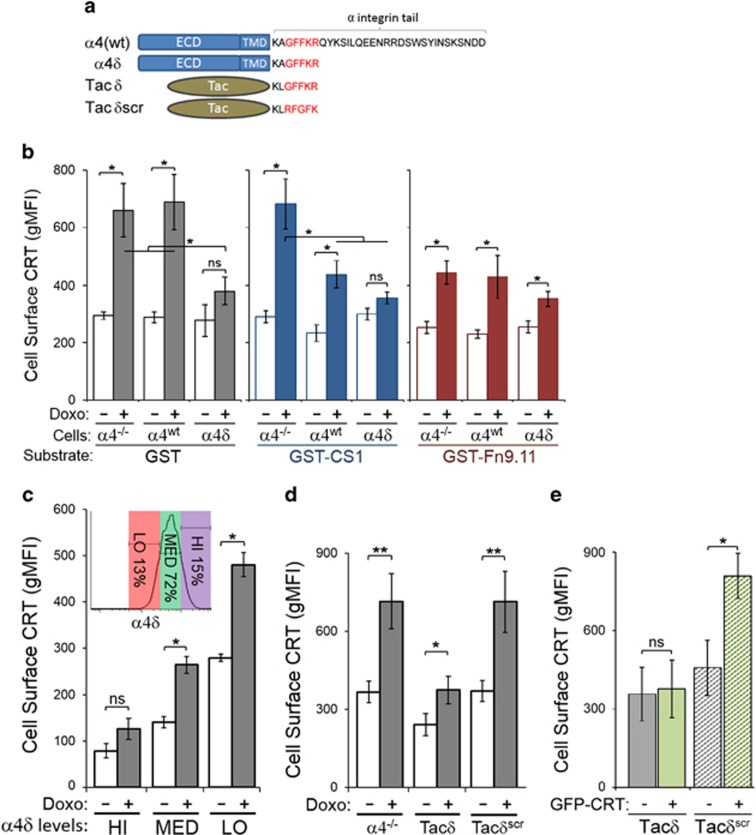
Expression and function of *α*-integrin reduces presentation of surface CRT. (**a**) Schematic of *α*-integrin constructs used in ‘rescue' study. *α*4(wt) is full-length *α*4; *α*4*δ* is truncated at the cytosolic tail; Tac is a carrier receptor fused to KLGFFKR (Tac*δ*) or scrambled KLRFGFK (Tac*δ*^scr^); ECD, extracellular domain, TMD, transmembrane domain. (**b**) Flow cytometry gMFI plots of surface CRT on *α*4^−/−^, *α*4^WT^ (*α*4^−/−^ reconstituted with *α*4^WT^) or *α*4*δ* cells (*α*4^−/−^ reconstituted with *α*4*δ*) that were plated on GST-CS1 (*α*4*β*1 ligand), GST-Fn9.11 (*α*5*β*1 ligand) or GST alone (no integrins engaged), untreated or treated with doxorubicin (Doxo). Plotted are the mean±S.D.; *n*=3; *P<0.05. (**c**) Polyclonal *α*4*δ* cells were stained for surface *α*4 and gated for low (LO), medium (MED) and high (HI) levels of *α*4*δ* expression (inset) as indicated to determine surface CRT levels when untreated or treated with doxorubicin. The flow cytometry gMFI plots are the mean±S.D.; *n*=3; *P<0.01; NS=not significant. (**d**) Flow cytometry gMFI plots of surface CRT on *α*4^−/−^, *α*4^−/−^/Tac*δ* and *α*4^−/−^/Tac*δ*^scr^ cells, untreated or treated with doxorubicin. Plotted are the mean±S.D.; *n*=3; **P<0.01, *P<0.05. Cells in (**c**–**d**) were assayed in suspension and not plated on any substrate. (**e**) Flow cytometry gMFI plots of surface CRT on Tac*δ* and Tac*δ*^scr^ cells, either untransfected or transfected to express GFP-CRT. Plotted are the mean±S.D.; *n*=3; *P<0.03, NS=not significant. Data shown in (**b**–**e**) are representative of three independently conducted experiments

We, and others, reported that *α*-integrins exhibit increased interaction with CRT upon cell adhesion to integrin substrates.^21, 23, 24^ This interaction is dependent on the GFFKR peptide motif found at the juxtamembrane cytosolic tail of *α*-integrins; thus, we speculated that *α*-integrin function may modulate surface CRT presentation. To address this, we assessed surface CRT levels for cells plated on integrin substrates. We used a Jurkat-derivative line lacking *α*4-integrins (*α*4^−/−^), and *α*4^−/−^ stably reconstituted with full-length *α*4 (*α*4^wt^), or with a cytoplasmic tail-truncated variant (*α*4*δ*) that terminates with the KxGFFKR cytosolic motif (Figure 3a). Previously, we showed that *α*4*δ*-expressing cells do not adhere to an *α*4*β*1-specific substrate, yet exhibit increased *α*4*δ* binding to CRT in an adhesion-independent manner.^23^

When plated on glutathione *S*-transferase (GST)-coated wells, a non-integrin engaging control substrate, doxorubicin-treated *α*4*δ* cells showed significantly lower surface CRT compared with *α*4^−/−^ and *α*4^wt^ cells (Figure 3b). When plated on CS1, a fibronectin-derived fragment that specifically engages *α*4*β*1-integrins,^30^ doxorubicin-treated *α*4^wt^ cells had lower surface CRT compared with *α*4^−/−^ cells (Figure 3b). When plated on Fn9.11, an RGD-containing fibronectin fragment that can engage multiple integrins (including *α*5*β*1-integrins expressed in all three cell lines),^23^ doxorubicin-treated *α*4^−/−^ and *α*4^wt^ cells had lower surface CRT compared with the same cells plated on GST. Importantly, *α*4*δ* cells exhibited low surface CRT under both adherent and non-adherent conditions, and this low level was comparable to *α*4^−/−^ and *α*4^wt^ cells under adherent conditions (Figure 3b).

As *α*4*δ* binds CRT in a constitutive manner,^23^ we postulated that the juxtamembrane GFFKR motif may sequester CRT in the cytosol and reduce CRT translocation to the cell surface. In this manner, we predicted that cells with more α4*δ* expression will have less surface CRT. Thus, we gated a polyclonal *α*4*δ*-expressing cell line for high, medium and low *α*4*δ* expression, and showed that surface CRT levels were inversely correlated with *α*4*δ* levels, both with and without doxorubicin treatment (Figure 3c). To determine if the juxtamembrane CRT-binding motif, GFFKR, is sufficient to inhibit surface CRT, we also assessed cells expressing the Tac carrier epitope fused to KLGFFKR (Tac*δ*) or the scrambled KLRFGFK (Tac*δ*^scr^) (Figure 3a).^23^

Similar to *α*4*δ* cells, doxorubicin-treated Tac*δ* cells had significantly reduced surface CRT when compared with control or Tac*δ*^scr^ cells (Figure 3d). Furthermore, expression of the cytosolic GFP-CRT resulted in increased surface CRT detected in control Tac*δ*^scr^ cells, but not in Tac*δ* cells (Figure 3e). Unlike *α*4*δ* or *α*4^wt^, Tac*δ* is a monomer that cannot heterodimerize with integrin-*β*1; thus, the reduced surface CRT observed for Tac*δ* cells is directly attributed to the GFFKR motif. These results demonstrated that doxorubicin-mediated surface CRT presentation was reduced for cells engaging integrin substrates. In contrast, cells expressing the juxtamembrane GFFKR motif (as *α*4*δ* or Tac*δ*) exhibited constitutively low levels of surface CRT in a manner that bypassed the requirement for cell adhesion.

### Cell adhesion-mediated reduction in surface CRT involves *β*1-integrins

**Figure 4:**
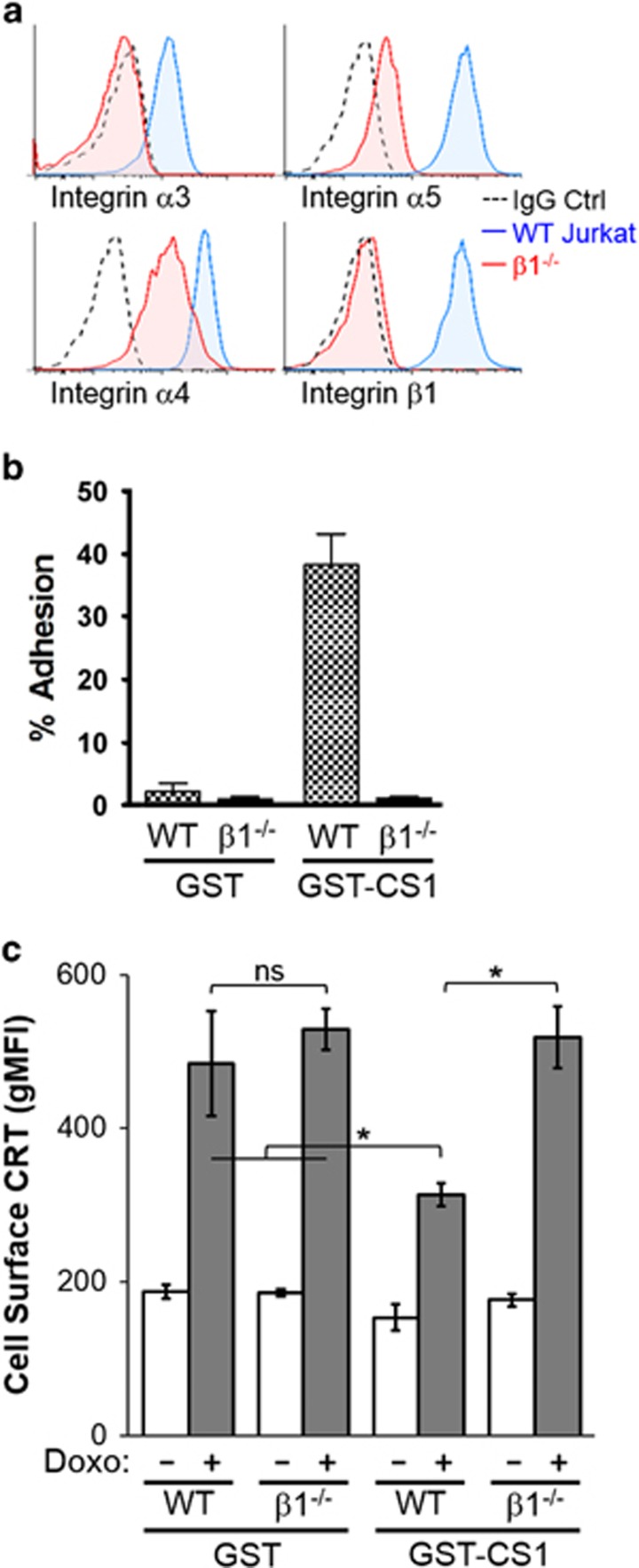
Loss of *β*1-integrin abolishes cell adhesion-mediated reduction in surface CRT. (**a**) Flow cytometry plots of WT and *β*1^−/−^ Jurkat cells showing relative expression of integrins *α*3, *α*4, *α*5 and *β*1. (**b**) Adhesion assay of WT and *β*1^−/−^ cells plated on GST- (control substrate) or GST-CS1- (*α*4*β*1 ligand) coated substrates. (**c**) Flow cytometry gMFI plots of surface CRT on WT and *β*1^−/−^ cells that were plated on GST or GST-CS1, untreated or treated with doxorubicin (Doxo). Plotted are the mean±S.D.; *n*=3; *P<0.01; NS=not significant. Data shown are representative of two independently conducted experiments. IgG, immunoglobulin G

As *β*1-integrin heterodimerizes with multiple *α*-integrins, loss of the *β*1-subunit yields corresponding decreases in the expression of multiple integrins. We assessed and found decreased expression of *α*3-, *α*4- and *α*5-integrins in *β*1^−/−^ cells compared with WT (Figure 4a). We also confirmed that *β*1^−/−^ cells failed to adhere to the *α*4*β*1-specific substrate CS1 (Figure 4b). Then, we assessed the functional contribution of *β*1-integrin on surface CRT presentation upon substrate adhesion. When plated on GST under non-adherent conditions, both *β*1^−/−^ and WT cells responded to doxorubicin treatment with comparable increases in surface CRT (Figure 4c). When plated on CS1 substrate to engage *α*4*β*1-integrins, WT cells exhibited a marked decrease, whereas *β*1^−/−^ cells exhibited no reduction in surface CRT compared with non-adherent conditions (Figure 4c). Thus, the evidence supports a modulatory role for integrin-mediated cell adhesion in decreasing surface CRT presentation for doxorubicin-treated cells.

### Antibody-induced *β*1-integrin activation inhibits surface CRT presentation

Cell adhesion is facilitated by activated integrins that take on the high-affinity ligand binding state and our results suggest that this state correlates with reduced surface CRT. Therefore, we tested if antibodies known to promote *β*1-integrin activation, such as 9EG7,^31^ could also downregulate surface CRT in the absence of cell adhesion.

**Figure 5:**
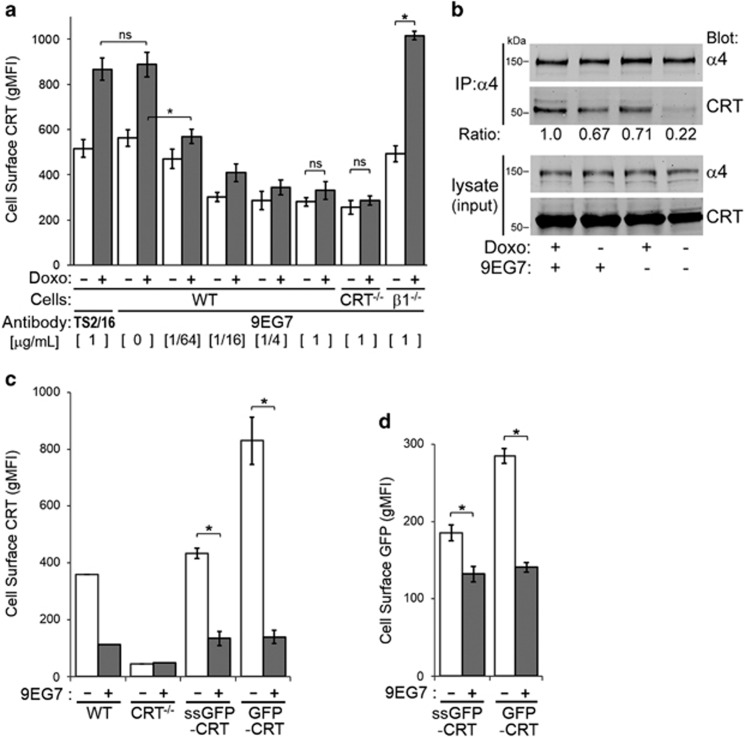
Activation of integrins with 9EG7 antibody reduces surface CRT levels. (**a**) Flow cytometry gMFI plots of surface CRT on suspension WT, CRT^−/−^ and *β*1^−/−^ cells, untreated or treated with doxorubicin (Doxo), and with the indicated concentrations of 9EG7 (*β*1-activating) or TS2/16 (*β*1-non-activating) antibodies. (**b**) *α*4-Integrins was immunoprecipitated (IP) from lysates of Jurkat cells that was untreated or treated in suspension with doxorubicin and 9EG7, as indicated, and analyzed by immunoblotting for CRT and *α*4. Densitometry analysis was performed to determine the CRT–*α*4 signal intensity ratio in the immunoprecipitates. (**c** and **d**) CRT^−/−^ cells were transfected to express ssGFP-CRT or GFP-CRT as indicated, and untreated or treated with 1 *μ*g/ml 9EG7 antibody. As shown are flow cytometry gMFI plots of surface CRT detected with (**c**) *α*-CRT or (**d**) *α*-GFP antibodies. Plotted are the mean±S.D.; *n*=3; *P<0.01. Absence of error bars indicate samples performed without replicates. Data shown are representative of three independently conducted experiments. NS, not significant

We found that 9EG7 binds to suspension Jurkat cells in a concentration-dependent manner (Supplementary S4). Then, we assayed surface CRT for cells treated with 9EG7 and/or doxorubicin to induce surface CRT. Remarkably, increasing concentrations of 9EG7 reduced surface CRT on WT cells to the low levels observed on CRT^−/−^ cells (Figure 5a). Importantly, incubation of 9EG7 with *β*1^−/−^ cells had no inhibitory consequence on doxorubicin-induced surface CRT, confirming the specificity of 9EG7 for *β*1-integrins. WT cells treated with the *β*1-specific but non-activating TS2/16 antibody also showed no inhibitory consequence on surface CRT. Notably, the 9EG7-mediated decrease of surface CRT in WT cells was observed for both doxorubicin-treated and non-treated conditions (Figure 5a), suggesting the generality of an integrin activation-mediated effect that did not require anthracycline induction of ER stress.

To gain insight on how integrin function can prevent surface presentation of CRT upon treatment with ICD inducers, we conducted integrin immunoprecipitation for doxorubicin-treated or untreated cells, and with and without 9EG7, as before. Doxorubicin effectively promoted an increased level of CRT that immunoprecipitated with *α*4-integrin, and this association was increased further for 9EG7-treated cells (Figure 5b). Furthermore, CRT^−/−^ cells expressing either the ER-targeted ssGFP-CRT or cytosol-targeted GFP-CRT treated with 9EG7, but not doxorubicin, resulted in similarly and significantly reduced levels of surface CRT and GFP detected (Figures 5c and d). This confirmed that 9EG7-mediated integrin activation can inhibit surface translocation of CRT from a predominantly cytosolic locale.

### Cell adhesion and 9EG7 reduces surface CRT presentation in various T-ALL

**Figure 6:**
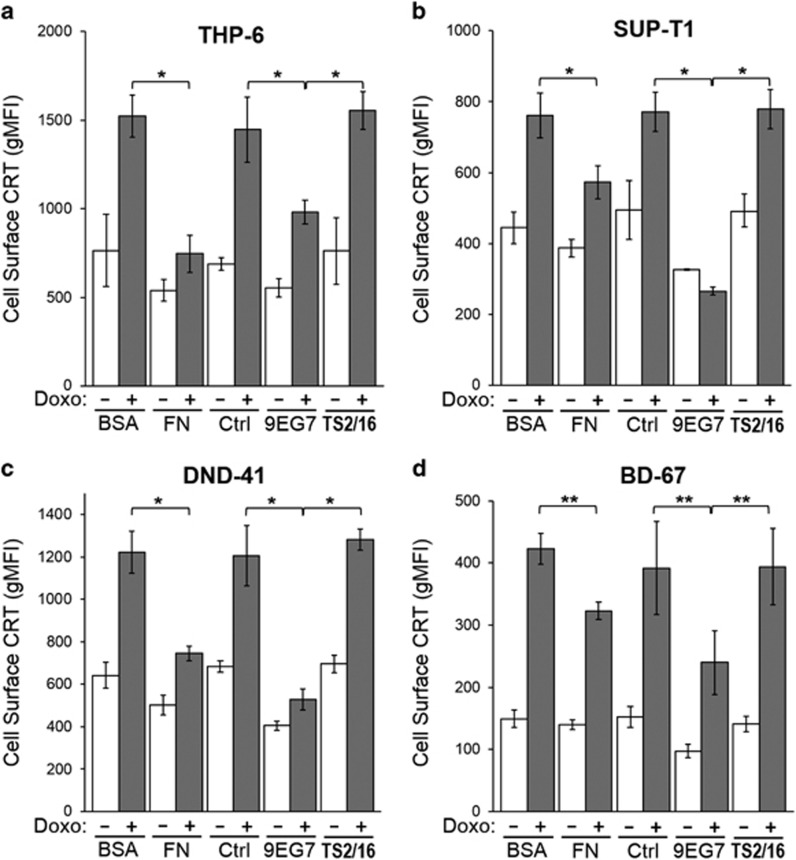
Cell adhesion or 9EG7 antibody treatment reduces surface CRT presentation in various T-ALL leukemias. Flow cytometry gMFI plots of surface CRT on various cells untreated or treated with doxorubicin (Doxo), and either plated on fibronectin (FN) or BSA, or incubated in suspension with either 1 *μ*g/ml 9EG7 or TS2/16 antibodies. Plotted are the mean±S.D.; *n*=3; *P<0.01; **P<0.05. As indicated, (**a**) THP-6, (**b**) SUPT-1 and (**c**) DND-41 are T-ALL cell lines, and (**d**) BD-67 is a murine xenograft-expanded primary human T-ALL leukemia. Data are representative of three independently conducted experiments for (**a**–**c**) and two experiments for (**d**). Ctrl, control

To determine if the suppression of surface CRT by stimulated integrin function occurred in other T-lymphoblasts, we repeated key assays using the human T-ALL cell lines THP-6, SUP-T1 and DND-41, as well as a primary human T-ALL, BD-67. As before, cells were treated with doxorubicin to stimulate surface CRT, plated on fibronectin-coated dishes to assess adhesion effects and/or treated in suspension with 9EG7 antibodies to assess integrin activation effects. In agreement with the Jurkat cell observations, doxorubicin-induced surface CRT was significantly reduced for cells adherent on fibronectin and for cells treated with 9EG7 when compared with their respective controls (Figures 6a–d).

### CRT released from the ER by drug treatment is bound by integrins in the cytosolic compartment

**Figure 7:**
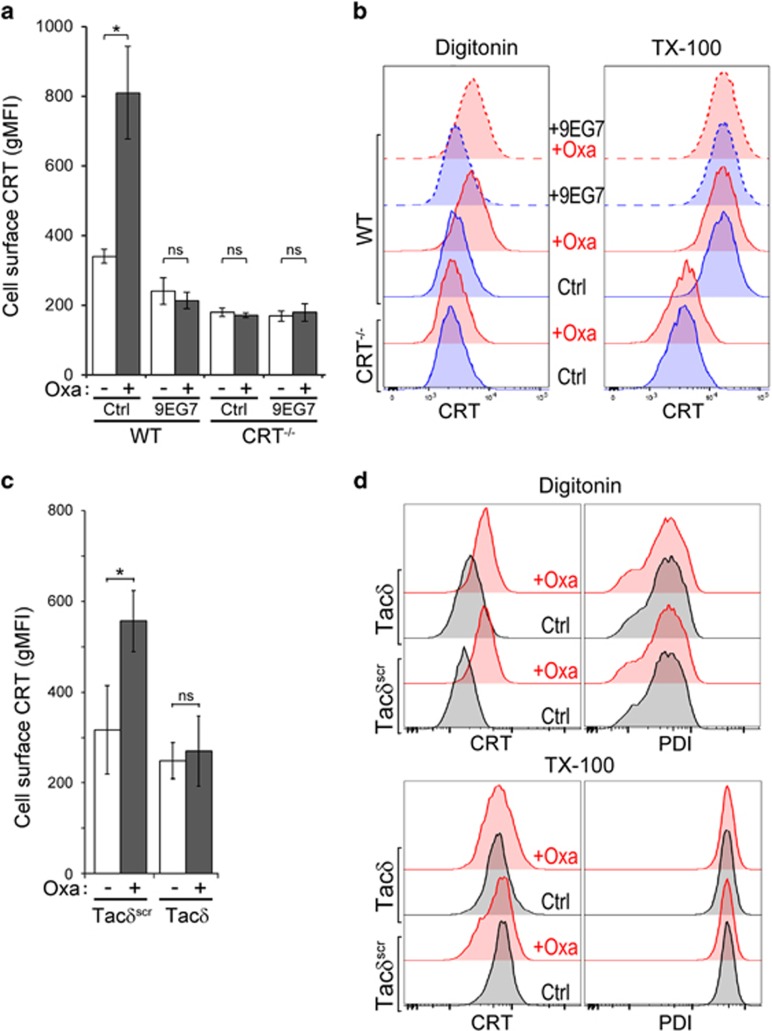
Drug-induced release of CRT from the ER is enriched in the cytosol. (**a**) Flow cytometry gMFI plots of surface CRT on Jurkat WT and CRT^−/−^ cells, untreated or treated with 1 *μ*g/ml 9EG7 antibody and/or 300 μM oxaliplatin (Oxa). Plotted are the mean±S.D.; *n*=3; *P<0.01; NS=not significant. (**b**) The indicated cells were untreated, or treated with oxaliplatin, 9EG7 or both, fixed in suspension and immunostained for CRT following partial permeabilization with digitonin, or full permeabilization with Triton X-100 (TX-100). As plotted is the flow cytometry analysis to compare CRT signal intensity. (**c**) Flow cytometry gMFI plots of surface CRT on Tac*δ* and Tac*δ*^scr^ cells, untreated or treated with 300 *μ*M oxaliplatin. Plotted are the mean±S.D.; *n*=3; *P<0.03; NS=not significant. (**d**) Similar to (**b**) but conducted with Tac*δ* and Tac*δ*^scr^ cells and costained for both CRT and PDI. Data are representative of two independently conducted experiments for (**a**) and (**c**), and for two replicates in two experiments for (**b**) and (**d**). Ctrl, control

As 9EG7 co-treatment inhibited surface CRT and increased CRT–integrin interaction (Figure 5), we postulated that drug treatment promoted release of ER-resident CRT into the cytosol where it is able to interact with the integrin cytosolic domain. Doxorubicin is fluorescent and incompatible for multicolor immunofluorescence imaging, thus we used oxaliplatin, a non-fluorescent and well-characterized ICD-inducing agent.^32^ First, we confirmed that surface CRT is induced in oxaliplatin-treated Jurkat cells, and that 9EG7 similarly inhibited surface CRT exposure (Figure 7a).

To enable differential immunostaining of cytosolic- and ER-resident CRT, we used partial permeabilization techniques using digitonin, *versus* full permeabilization using TX-100.^10^ WT cells show robust staining for PDI and CRT when fully permeabilized with Triton X-100, but not when treated with a digitonin concentration that enables permeabilization of the plasma membrane but not the ER (Supplementary S5). We observed a low but significant level of digitonin-permeabilized CRT in oxaliplatin-treated WT cells, suggesting staining of CRT other than the ER pool.

To quantify the results, we repeated the assay by flow cytometry, as this allows assessment of total fluorescence that is not limited by optical sectioning (Figure 7b). Cells were treated with oxaliplatin, 9EG7 or both. Regardless of treatment conditions, TX-100-permeabilized WT cells exhibited comparable staining for CRT, indicating no significant changes of total CRT by the various treatments. In contrast, CRT staining in digitonin-permeabilized and oxaliplatin-treated WT cells was significantly elevated over oxaliplatin-untreated cells, regardless of 9EG7 co-treatment (Figure 7b). In a similar manner, cells expressing Tac*δ* exhibited no increase in surface CRT upon oxaliplatin treatment, even though cytosolic CRT was significantly increased (Figures 7c and d).

Taken together, our results show that cells treated with the integrin activator 9EG7, or expressing the minimal *α*-integrin GFFKR, have markedly reduced surface CRT when challenged with an ICD inducer, even though CRT was elevated in the extra-ER, cytosolic compartment. This cytosolic pool of CRT is also observed as an increased interaction with *α*4-integrin. As *α*-integrins interact with CRT via the cytosolic GFFKR motif, our results support a model where activated integrins bind cytosolic CRT and prevent its translocation to the cell surface.

### 9EG7-mediated reduction of surface CRT decreases phagocytic engulfment

**Figure 8:**
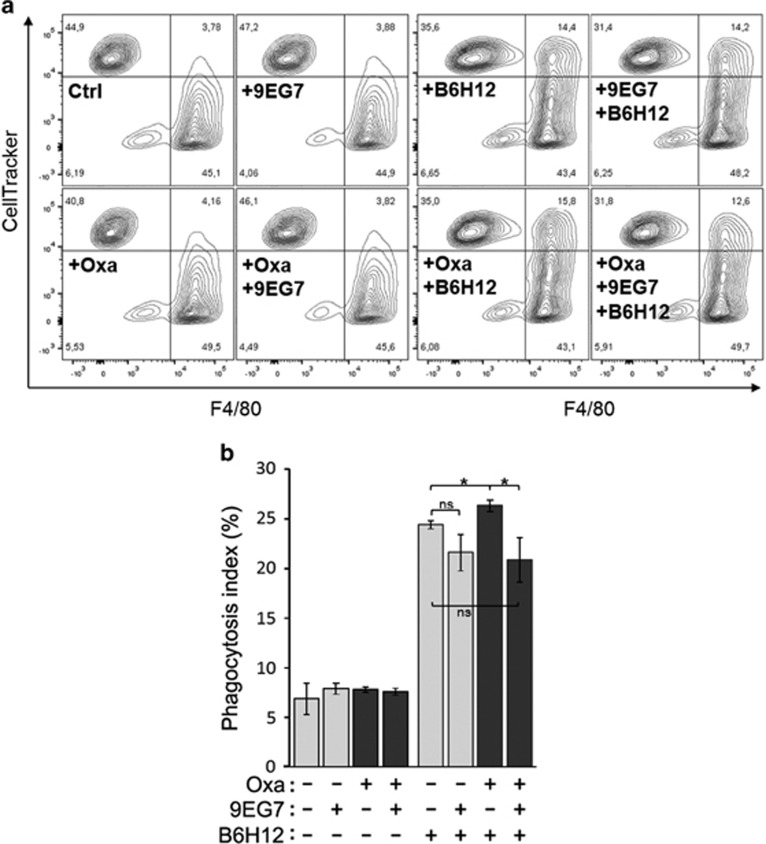
9EG7 antibody treatment of Jurkat cells reduces their phagocytosis by macrophages. As indicated, Jurkat cells were untreated or pretreated with 9EG7 (*β*1-activating) or B6H12 (*α*-CD47) antibodies, and with or without oxaliplatin (Oxa). Pretreated cells were then coincubated with primary mouse macrophages for 2 h and phagocytosis determined by flow cytometry as described in Materials and Methods. (**a**) Phagocytosis assay showing representative flow cytometry plots. F4/80 labels macrophages, whereas CellTracker labels Jurkat T-lymphoblasts. (**b**) Phagocytosis index (%) is calculated as 100 × (CellTracker^+^, F4/80^+^ macrophages/total macrophages) and plotted as shown are the mean±S.D.; *n*=3; *P<0.02; NS=not significant. Data shown are representative of three independently conducted experiments. Ctrl, control

To determine if the integrin-mediated inhibition of surface CRT mediated by ICD inducers have a measurable outcome on target cell engulfment by professional phagocytes, we performed a phagocytosis assay using macrophages. Jurkat target cells were treated with oxaliplatin, 9EG7 and combinations thereof. To counter Jurkat cells' high levels of cluster of differentiation 47 (CD47) expression,^33^ a known inhibitory receptor for macrophage-mediated phagocytosis,^6, 34^ cells were also coincubated with the CD47-neutralizing antibody, B6H12. The treated Jurkat cells were coincubated with macrophages, and the labeled cell mixture was analyzed by flow cytometry to delineate and quantitate single-positive (Jurkat or macrophage) and double-positive (Jurkat/s engulfed by macrophage) populations (Figure 8a). The data for all conditions are plotted as a phagocytosis index (Figure 8b).

In agreement with similar assays conducted by others,^35, 36^ we found that CD47 neutralization with B6H12 antibody was necessary to reveal a three- to fourfold higher rate of phagocytosis (Figure 8). Under these conditions, macrophage-mediated phagocytosis was significantly increased for oxaliplatin-treated compared with untreated Jurkat cells. Importantly, co-treatment with 9EG7 antibody resulted in reduced phagocytosis of cells treated with or without oxaliplatin. Taken together, our results show that the integrin-activating 9EG7 antibody can suppress surface presentation of CRT in T-lymphoblasts to levels that reduced its ability to be engulfed by macrophages.

## Discussion

Our study is the first to describe a regulatory role for *α*-integrin function in cell surface CRT presentation. By generating and reconstituting CRT^−/−^ T-lymphoblasts with GFP-tagged CRT that was either expressed in the cytosol or enriched within the ER lumen, afforded the opportunity to probe the subcellular source for surface CRT resulting from an ICD inducer. The increased surface CRT presentation following doxorubicin treatment of cells expressing the ER-targeted ssGFP-CRT is consistent with earlier reports, suggesting that the path to the cell surface requires induction of ER stress and CRT release from its highly enriched localization within the ER lumen.^8, 37, 38^ This increase was detectable with either CRT or GFP antibodies, indicating that the translocation involved the intact fusion protein. When expressed within the cytosol without transiting the ER, GFP-CRT presented at high levels on the cell surface in a manner that could not be stimulated further with doxorubicin, thus effectively bypassing the requirement for ER stress. Several hypotheses have been put forth on the pathway for CRT transit from the ER to the cell surface.^39, 40, 41, 42^ Our findings support a mechanism that involves CRT release from the stressed ER to the cytosol via the ubiquitination pathway,^43, 44, 45, 46, 47^ following which CRT translocates to the surface.

At the plasma membrane, *α*-integrins appear to regulate surface CRT presentation. The C-terminal cytosolic tail encoded by all *α*-integrins has a conserved GFFKR peptide motif required for heterodimer stabilization with *β*-integrin.^48, 49^ Structural studies has revealed that the *α*- and *β*-integrin tails become physically separated upon integrin activation,^50^ facilitating binding of proteins to the tails. We, and others, have reported that CRT interacts with integrins via this motif in an adhesion-dependent manner.^21, 23, 24^ We adopted several strategies to test the assumption that increased integrin activation leads to increased intracellular CRT–integrin interaction, thus reducing ‘free' cytosolic CRT able to translocate to the extracellular surface. As a physiologically relevant stimulus, we show that T-lymphoblast adhesion to an integrin substrate significantly reduces surface CRT in cells treated with ICD inducers. Similarly, suspension cells treated with the *β*1-integrin-activating antibody, 9EG7, elicited a greater suppressive effect on surface CRT when compared with adhesion, in most cases reducing surface CRT on WT cells to the levels observed for CRT^−/−^ cells. Combined with the observations that 9EG7 treatment, or expression of the juxtamembrane-anchored cytosolic GFFKR motif, suppressed the appearance of surface CRT for CRT^−/−^ cells expressing the cytosol-targeted GFP-CRT, we suggest that a cytosolic pool of CRT exists in T-lymphoblasts and that activated integrins interact primarily with cytosolic CRT.

Previously, we showed that expression of a transmembrane-anchored *α*-integrin tail consisting of only the GFFKR motif (as *α*4*δ* or Tac*δ*) resulted in increased interaction with CRT in an adhesion-independent manner.^23^ In this study, we show that *α*4*δ* and Tac*δ* cells exhibit constitutively lower levels of surface CRT, again in an adhesion-independent manner. Furthermore, we correlated higher *α*4*δ* expression with lower surface CRT, suggesting that the truncation may have enabled binding of CRT to the now more accessible juxtamembrane GFFKR motif. A greater level of CRT associated with *α*4-integrins when cells were treated with an ICD inducer, likely resulting from a marked increase in cytosolic CRT released from the ER, now able to interact with *α*-integrin tails. We suggest the increased CRT–integrin interaction effectively sequestered CRT within the cytosol and prevented its translocation to the extracellular surface. Beyond the GFFKR-conserved motif, *α*-integrin tails share little sequence homology;^20^ thus, an interesting question for future studies would be to compare surface CRT levels for cells specifically engaging various *α*-integrins.

As a DAMP expressed on the surface of cells undergoing ICD, surface CRT is crucial for the clearance of tumor cells by the host's immune system mediated by professional phagocytes.^3, 6, 7^ Therefore, cell physiological events contributing to reduced surface CRT expression may lead to reduced antitumor responses resulting from ICD-based chemotherapy. The tumor microenvironment such as the bone marrow stroma may provide these protective stimuli in the form of adhesion substrates and stimulatory chemokines that promote integrin activation.^18^ Thus, effective chemotherapy using ICD inducers may benefit from coadministration with integrin function blocking therapeutic antibodies.

## Materials and Methods

### Human T-ALL cell lines and cells

Jurkat cells was from ATCC (Manassas, VA, USA). The Jurkat-derivative *α*4^−/−^, *α*4^wt^, *α*4*δ*, Tac*δ* and Tac*δ*^scr^ was described previously.^23^ Dr Shimizu provided the *β*1^−/−^ strain.^51^ Dr. Weng provided THP-6, SUP-T1 and DND-41. Cells were cultured at 37 °C, 5%CO_2_ in complete RPMI (cRPMI is RPMI-1640, 10% FBS, pen-strep and nonessential amino acids; Thermo Fisher, Waltham, MA, USA). Some cells were culture adapted in Cell-Ess-supplemented media (Essential Pharmaceuticals, Ewing, NJ, USA) in place of FBS, as per the manufacturer's instructions. Cell transfection was by nucleofection (Lonza, Walkersville, MD, USA).

CRT^−/−^ cells was generated by the CRISPR-Cas9 methodology^25^ using 5′-CGAGCCTGCCGTCTACTTCA-3′ *CALR* guide DNA and plasmid pX330. Following nucleoporation and sorting, CRT^−/−^ clones were identified by fluorescence immunostaining for CRT and confirmed by sequencing the targeted genomic loci. Clone hCRT1-3 was used to generate the data shown, with the major phenotypes reproduced in three independent clones.

Primary T-ALL (BD-67) was obtained from the BC Children's Hospital Biobank (Vancouver, BC, Canada) with ethics approval from the BC Women's and Children's Hospital institutional review board (H12-03216). Relapsed bone marrow aspirate was injected via tail vein into NOD-scid/IL-2R*γ*-null (NSG) mice (Jackson Laboratory, Bar Harbor, ME, USA). Mice were monitored for human leukemia engraftment by flow cytometric analysis of peripheral blood. Those with high leukemia burden were killed and their spleens (~80% CD45^+^ lymphoblasts) immediately sourced for primary T-ALL cells.

### Flow cytometry

FACSCanto, LSRFortessa and Accuri C6 was used for analytical work and FACSAria (BD, Mississauga, ON, Canada) for cell sorting. Postacquisition analysis was carried out using FlowJo (Tree Star, Ashland, OR, USA).

### Antibodies

The antibodies used for flow cytometric detection of surface antigens were: *α*4- (9F10) and *α*5-integrin (NKI-SAM-1; BioLegend, San Diego, CA, USA); *β*1-integrin (sc-53711; Santa Cruz, Dallas, TX, USA); CRT (ab2907) and ERp57 (ab10287; Abcam, Toronto, ON, Canada); F4/80 (BM8) and MHC-I (W6/32, BioLegend); GFP (GF28R; Thermo Fisher). The antibodies used for immunofluorescence microscopy were: CRT (ab2907) and PDI (ab2792; Abcam). Those used for immunoprecipitation and immunoblotting were: *α*4-integrin (HP2/1), CRT (PA3-900, Thermo Fisher) and GAPDH (FF26A/F9; BioLegend). And, those used for integrin activation and phagocytosis assays were: *β*1-integrin (9EG7; Thermo Fisher) and CD47 (B6H12; BD).

### Plasmids

GFP-CRT is a N-terminal GFP fusion to human CRT (amino acids 18–417), provided by Dr. Eggleton.^44^ To reconstitute ER targeting, the ss of CRT (amino acids 1–17) was fused N-terminal to GFP-CRT, producing ssGFP-CRT.

### Adhesion substrates

Purified recombinant proteins, GST, GST-CS1, GST-Fn9.11 and human plasma fibronectin was described previously.^23^ CS1 (connecting segment 1)^30^ and Fn9.11 (repeats 9–11)^52^ are peptide fragments derived from fibronectin. Bovine serum albumin (BSA) and GST was used as a non-adherent control substrate. Culture dishes (Corning, Corning, NY, USA) were coated with 40 *μ*g/ml of fibronectin, GST-CS1, GST-Fn9.11, GST or BSA in phosphate-buffered saline (PBS). Adhesion assays were conducted as described previously.^33^

### Cell surface CRT assays

A total of 10^6^ cells per ml in cRPMI were treated with 4 *μ*g/ml doxorubicin (Sigma-Aldrich, St. Louis, MO, USA) for 4 h, or 300 μM oxaliplatin (Tocris, Minneapolis, MN, USA) for 2 h at 37 °C. Where applicable, cells were serum-starved in blank RPMI for 24 h before drug treatments. Some cells were nucleoporated with GFP-CRT or ssGFP-CRT 24 h before drug treatments and the transfected population analyzed by gating for GFP-positive cells. For adhesion assays, cells were seeded on substrate-coated wells 1h before drug treatments. Integrin-binding antibodies was added to cells 15 min prior, and remain throughout the drug treatment. Surface CRT or GFP-CRT levels was measured by flow cytometry analysis of *α*-CRT or *α*-GFP antibody-labeled cells using 633 nm excitation. Only the non-apoptotic (Annexin V-negative) population was gated for geometric mean fluorescence intensity (gMFI) calculation.

### Partial permeabilization and immunostaining for CRT

Following treatments, cells were fixed in 3.7% formaldehyde/PBS for 15 min and divided into two groups treated with 0.1% Triton X-100/PBS or 25 *μ*g/ml digitonin/PBS (Acros, Waltham, MA, USA) for 5 min. Following washes, cells were immunostained for CRT and analyzed by flow cytometry. We optimized digitonin concentrations based on the ability to maintain ER integrity that precluded staining for PDI, an ER marker.^10^

### Fluorescence microscopy

Cells plated onto fibronectin-coated coverslips was fixed in 3.7% formaldehyde/PBS, permeabilized in 0.1% Triton X-100/PBS and immunostained accordingly. Images were acquired on an Olympus IX81 (Olympus Canada, Richmond Hill, ON, Canada) microscope equipped with a 60 × NA1.35 oil-immersion objective. Postacquisition processing was conducted using ImageJ (https://imagej.nih.gov/ij/). Some cells were digitonin permeabilized as described.

### Immunoprecipitation and immunoblot analysis

Lysates were prepared in PN buffer (10 mM PIPES, 50 mM NaCl, 150 mM sucrose, 50 mM NaF, 40 mM Na_4_P_2_O_7_·10H_2_O, 1 mM CaCl_2_, 1 mM MgCl_2_, 1% Triton X-100, complete protease inhibitors; Roche, St. Louis, MO, USA). For *α*4 immunoprecipitation, 1 mg lysate was incubated with 1 μg HP2/1 antibody, and precipitated with protein A/G-Sepharose (Pierce, Waltham, MA, USA). Western blot analyses was described previously.^23^ Densitometry analyses was performed on ImageJ.

### Phagocytosis assay

Femura and tibiae bone marrow aspirates of 8-week-old C57BL/6 mice were plated at 5 × 10^5^ cells per ml for 4 h in complete IMDM (cIMDM, 10% FBS, pen-strep). Non-adherent cells were replated in cIMDM with 10 ng/ml murine MCSF (StemCell, Vancouver, BC, Canada) for 10 days. Adherent cells (macrophages) from 10-day cultures were >95% Mac-1^+^ and F4/80^+^. Macrophages were lifted and starved for 1 h in blank IMDM (bIMDM) before coincubating with target cells.

Target Jurkat cells were prelabeled with CellTracker (Invitrogen, Carlsbad, CA, USA) as per the manufacturer's instructions and incubated with 1 *μ*g/ml 9EG7 (*β*1-activating antibody) for 4 h at 37 °C. As applicable, cells were coincubated for the final 2 h with 7 *μ*g/ml *α*-CD47 (B6H12) and/or 300 *μ*M oxaliplatin. Phagocytosis was initiated by coplating 2.4 × 10^5^ macrophages with 1.2 × 10^6^ washed Jurkat cells in 24-well plates for 2 h in bIMDM at 37 °C. Macrophages were stained with F4/80 antibodies, and total cell mixture analyzed by flow cytometry. Phagocytosis calculation: % Phagocytosis=100 × (CellTracker^+^, F4/80^+^ macrophages/total macrophages).

### Statistical analysis

*P*-values was calculated with the Student's *t*-test. Error bars are the standard deviation values obtained from at least three treatment replicates conducted within an experiment. All data shown are representative of two to three independently conducted experiments, and indicated as such.

## Abbreviations

ALL: acute lymphoblastic leukemia
ATP: adenosine triphosphate
CD47: cluster of differentiation 47
CRT: calreticulin
CRT^−/−^: calreticulin null
DAMP: damage-associated molecular pattern
ER: endoplasmic reticulum
ERp57: endoplasmic reticulum-resident protein 57
FBS: fetal bovine serum
GFP: green fluorescent protein
GST: glutathione *S*-transferase
ICD: immunogenic cell death
MHC: major histocompatibility complex
PDI: protein disulfide isomerase
ss: signal sequence
WT: wild type
