# Supplementary material for: α-Integrin expression and function modulates presentation of cell surface calreticulin
Source: Cell Death Dis. 2016 Jun 16;7(6):e2268–. doi: 10.1038/cddis.2016.176 (PMC5143402; doi:10.1038/cddis.2016.176)
Supplement: Supplementary Information [file cddis2016176x1.doc]

**Figured Legends**

**Figure 1.** **Doxorubicin treatment induces cell surface CRT expression in Jurkat T-cells.**

**(a)** Western blot analysis of lysates from Jurkat wildtype (WT) and CRISPR-Cas9 generated CRT^-/-^ cells. **(b)** Sequencing of *CALR* genomic loci showing single nucleotide insertion (red triangle) occurring at 68 bp from the predicted start codon and -3 bp from the PAM recognition motif (green bar). The frame shift mutated variant encodes for a predicted 58 amino acid protein product due to a premature termination codon. **(c)** Representative flow cytometry plots of surface CRT comparing WT and CRT^-/-^ cells cultured in 10% FBS-supplemented RPMI, untreated or treated with doxorubicin (Doxo). **(d)** Flow cytometry geometric mean fluorescence intensity (gMFI) plots of surface CRT for the indicated cells cultured in either 10% FBS-supplemented RPMI, or serum-starved for 24 hr (0% FBS), and untreated or treated with doxorubicin. Plotted are the Mean ± SD; n=3; **p*<0.01; ***p*<0.05. **(e)** Representative flow cytometry plots of surface CRT comparing WT and CRT^-/-^ cells cultured in 10% FBS media or in 0.3%FBS/9.7% Cell Essential (serum replacement) media, untreated or treated with doxorubicin. **(f)** Flow cytometry gMFI plots of surface CRT as described in (e). Plotted are the Mean ± SD; n=3; **p*<0.001; ***p*<0.05; ns=not significant. Data shown in (c-f) are representative of 3 independently conducted experiments.

**Figure 2.** **Doxorubicin-mediated increase in surface calreticulin requires ER-resident calreticulin.**

**(a)** Schematic of wildtype CRT, GFP-CRT (cytosol-targeted) and ssGFP-CRT (ER-targeted). **(b)** The indicated cells were fixed, permeabilized with Triton TX-100, and immuno-stained as follows. Left Panels: Immunofluorescence images of endogenous CRT (green) and PDI (red) in Jurkat WT and CRT^-/-^ cells. Right Panels: CRT^-/-^ cells transiently transfected to express ssGFP-CRT or GFP-CRT (green) were stained for PDI (red). First column shows color merged images. Bars: 5 µm. **(c)** Flow cytometry gMFI plots of surface CRT on live WT, CRT^-/-^, and CRT^-/-^ cells expressing ssGFP-CRT or GFP-CRT, untreated or treated with doxorubicin. **(d)** Same as (c) but cells were stained with antibodies targeting cell surface GFP instead of CRT. Inset is a representative flow cytometry plot of Control non-transfected cells, and cells transfected to express GFP-CRT or GFP alone, which was stained for surface GFP with antibodies. Plotted are the Mean ± SD; n=3; **p*<0.01; ns=not significant. Data shown in (b-d) are representative of 3 independently conducted experiments.

**Figure 3.** **Expression and function of α-integrin reduces presentation of surface calreticulin.**

**(a)** Schematic of α-integrin constructs used in ‘rescue’ study. α4(wt) is full length α4; α4δ is truncated at the cytosolic tail; Tac is a carrier receptor fused to KLGFFKR (Tacδ) or scrambled KLRFGFK (Tacδ^scr^); ECD-Extracellular domain, TMD-Transmembrane domain. **(b)** Flow cytometry gMFI plots of surface CRT on α4^-/-^, α4^WT^ (α4^-/-^ reconstituted with α4^WT^) or α4δ cells (α4^-/-^ reconstituted with α4δ) that were plated on GST-CS1 (α4β1-ligand), GST-Fn9.11 (α5β1-ligand) or GST alone (no integrins engaged), untreated or treated with doxorubicin. Plotted are the Mean ± SD; n=3; **p*<0.05. **(c)** Polyclonal α4δ cells were stained for surface α4 and gated for low, medium and high levels of α4δ expression (inset) as indicated to determine surface CRT levels when untreated or treated with doxorubicin. The flow cytometry gMFI plots are the Mean ± SD; n=3; **p*<0.01; ns=not significant. **(d)** Flow cytometry gMFI plots of surface CRT on α4^-/-^, α4^-/-^/Tacδ and α4^-/-^/Tacδ^scr^ cells, untreated or treated with doxorubicin. Plotted are the Mean ± SD; n=3; ***p*<0.01, ******p*<0.05. Cells in (c-d) were assayed in suspension and not plated on any substrate. **(e)** Flow cytometry gMFI plots of surface CRT on Tacδ and Tacδ^scr^ cells, either untransfected or transfected to express GFP-CRT. Plotted are the Mean ± SD; n=3; **p*<0.03, ns=not significant. Data shown in (b-e) are representative of 3 independently conducted experiments.

**Figure 4.** **Loss of β1-integrin abolishes cell adhesion-mediated reduction in surface CRT.**

**(a)** Flow cytometry plots of WT and β1^-/-^ Jurkat cells showing relative expression of integrins α3, α4, α5 and β1. **(b)** Adhesion assay of WT and β1^-/-^ cells plated on GST (control substrate) or GST-CS1 (α4β1-ligand) coated substrates. **(c)** Flow cytometry gMFI plots of surface CRT on WT and β1^-/-^ cells that were plated on GST or GST-CS1, untreated or treated with doxorubicin. Plotted are the Mean ± SD; n=3; ******p*<0.01; ns=not significant. Data shown are representative of 2 independently conducted experiments.

**Figure 5. Activation of integrins with 9EG7 antibody reduces surface CRT levels.**

**(a)** Flow cytometry gMFI plots of surface CRT on suspension WT, CRT^-/-^ and β1^-/-^ cells, untreated or treated with doxorubicin, and with the indicated concentrations of 9EG7 (β1-activating) or TS2/16 (β1 non-activating) antibodies. **(b)** α4-integrins was immunoprecipitated from lysates of Jurkat cells that was untreated or treated in suspension with doxorubicin and 9EG7, as indicated, and analyzed by immunoblotting for CRT and α4. Densitometry analysis was performed to determine the CRT:α4 signal intensity ratio in the immunoprecipitates. **(c – d)** CRT^-/-^ cells were transfected to express ssGFP-CRT or GFP-CRT as indicated, and untreated or treated with 1µg/mL 9EG7 antibody. As shown are flow cytometry gMFI plots of surface CRT detected with **(b)** α-CRT or **(c)** α-GFP antibodies. Plotted are the Mean ± SD; n=3; ******p*<0.01. Absence of error bars indicate samples performed without replicates. Data shown are representative of 3 independently conducted experiments.

**Figure 6. Cell adhesion or 9EG7-antibody treatment reduces surface CRT presentation in various T-ALL leukemias.**

Flow cytometry gMFI plots of surface CRT on various cells untreated or treated with doxorubicin, and either plated on fibronectin (FN) or BSA, or incubated in suspension with either 1µg/mL 9EG7 or TS2/16 antibodies. Plotted are the Mean ± SD; n=3; ******p*<0.01; *******p*<0.05. As indicated, **(a)** THP-6 **(b)** SUPT-1 **(c)** DND41 are T-ALL cell lines, and **(d)** BD67 is a murine xenograft-expanded primary human T-ALL leukemia. Data are representative of 3 independently conducted experiments for (a-c) and 2 experiments for (d).

**Figure 7. Drug-induced release of CRT from the ER is enriched in the cytosol.**

**(a)** Flow cytometry gMFI plots of surface CRT on Jurkat WT and CRT^-/-^ cells, untreated or treated with 1µg/mL 9EG7 antibody and/or 300µM oxaliplatin. Plotted are the Mean ± SD; n=3; ******p*<0.01; ns=not significant. **(b)** The indicated cells were untreated, or treated with oxaliplatin, 9EG7 or both, fixed in suspension and immuno-stained for CRT following partial permeabilization with digitonin, or full permeabilization with TX-100. As plotted is the flow cytometry analysis to compare CRT signal intensity. **(c)** Flow cytometry gMFI plots of surface CRT on Tacδ and Tacδ^scr^ cells, untreated or treated with 300µM oxaliplatin. Plotted are the Mean ± SD; n=3; ******p*<0.03; ns=not significant. **(d)** Similar to (b) but conducted with Tacδ and Tacδ^scr^ cells and co-stained for both CRT and PDI. Data are representative of 2 independently conducted experiments for (a) and (c), and for 2 replicates in 2 experiments for (b) and (d).

**Figure 8.** **9EG7 antibody treatment of Jurkat cells reduces their phagocytosis by macrophages.**

As indicated, Jurkat cells were untreated or pretreated with 9EG7 (β1-activating) or B6H12 (α-CD47) antibodies, and with or without oxaliplatin. Pretreated cells were then co-incubated with primary mouse macrophages for 2 hr and phagocytosis determined by flow cytometry as described in Methods. **(a)** Phagocytosis assay showing representative flow cytometry plots. F4/80 labels macrophages, while CellTracker labels Jurkat T-lymphoblasts. **(b)** Phagocytosis index (%) is calculated as 100*(CellTracker^+^, F4/80^+^ macrophages/ total macrophages) and plotted as shown are the Mean ± SD; n=3; ******p*<0.02; ns=not significant. Data shown are representative of 3 independently conducted experiments.

**Supplemental S1**

Flow cytometry geometric Mean Fluorescence Intensity (gMFI) plots of surface CRT on WT and CRT^-/-^ cells cultured in 10% FBS media or serum-starved (0% FBS) for 24 hr. Following serum-starvation, cells were re-incubated for 2 hr in FBS-supplemented media at the indicated levels prior to cell surface staining with CRT antibody. Plotted are the Mean ± SD; n=3; **p*<0.01. Absence of error bars indicates samples conducted without replicates.

**Supplemental S2**

Flow cytometry histograms of MHC class I expression on live WT, CRT^-/-^, and CRT^-/-^ cells expressing ssGFP-CRT or GFP-CRT. Plotted data is representative of 3 independently conducted replicates.

**Supplemental S3**

Flow cytometry gMFI plots of surface ERp57 on live WT, CRT^-/-^, and CRT^-/-^ cells expressing ssGFP-CRT or GFP-CRT, untreated or treated with doxorubicin. Plotted are the Mean ± SD; n=3; **p*<0.02.

**Supplemental S4**

Flow cytometry gMFI plots of WT and β1^-/-^ cells treated with the indicated concentrations of 9EG7 (β1-activating) antibodies.

**Supplemental S5**

Untreated and oxaliplatin-treated cells were fixed in suspension, and either partially permeabilized with digitonin, or fully permeabilized with Triton X-100. Cells were then immunostained for CRT and PDI, and imaged under the same exposure settings. Shown are representative images of cells for each treatment group.

**Supplemental Table 1**

Extracellular ATP release mediated by doxorubicin treatment was measured for WT and CRT^-/-^ cells according to manufacturer’s instructions (Promega ELITEN). No significant ATP release was measured for cells treated with 4μg/mL doxorubicin for 4hr, or 300μM oxaliplatin for 2hr. As positive controls for ATP release, cells were treated with 0.2μg/mL doxorubicin, or 1.5μM oxaliplatin for 24hr. Values are the Mean ± SD; n=3.

| **Cells** | **Treatment** | **Extracellular ATP**  **(M, Mean ± SD)** |
| --- | --- | --- |
| WT | None, 4 hr | 0.6 x 10^-14^ ± 0.3 x 10^-14^ |
| CRT^-/-^ | None, 4 hr | 1.3 x 10^-14^ ± 1.2 x 10^-14^ |
| WT | 4μg/mL doxorubicin, 4hr | 0.7 x 10^-14^ ± 0.5 x 10^-14^ |
| CRT^-/-^ | 4μg/mL doxorubicin, 4hr | 0.7 x 10^-14^ ± 0.4 x 10^-14^ |
| WT | 300μM oxaliplatin, 2hr | 4.1 x 10^-14^ ± 4.0 x 10^-14^ |
| CRT^-/-^ | 300μM oxaliplatin, 2hr | 1.5 x 10^-14^ ± 1.0 x 10^-14^ |
|  |  |  |
| WT | None, 24 hr | 2.2 x 10^-10^ ± 0.7 x 10^-10^ |
| CRT^-/-^ | None, 24 hr | 3.5 x 10^-10^ ± 1.5 x 10^-10^ |
| WT | 0.2μg/mL doxorubicin, 24hr | 69.3 x 10^-10^ ± 22.5 x 10^-10^ |
| CRT^-/-^ | 0.2μg/mL doxorubicin, 24hr | 67.2 x 10^-10^ ± 15.7 x 10^-10^ |
| WT | 1.5μM oxaliplatin, 24hr | 41.7 x 10^-10^ ± 21.7 x 10^-10^ |
| CRT^-/-^ | 1.5μM oxaliplatin, 24hr | 35.1 x 10^-10^ ± 14.7 x 10^-10^ |
