# Supplementary figures and images for: α-Integrin expression and function modulates presentation of cell surface calreticulin
Source: Cell Death Dis. 2016 Jun 16;7(6):e2268–. doi: 10.1038/cddis.2016.176 (PMC5143402; doi:10.1038/cddis.2016.176)

## Supplementary S1

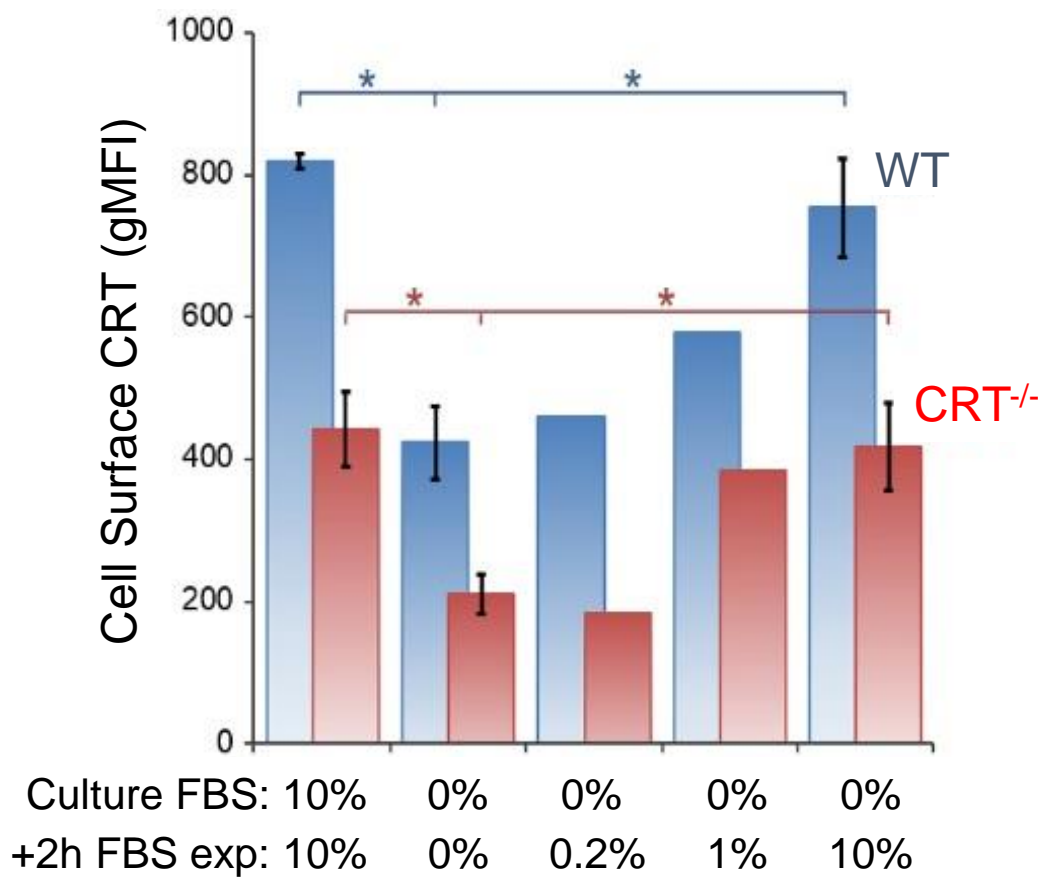

Supplement: Supplementary S1 [file cddis2016176x2.pdf]

## Supplementary S2

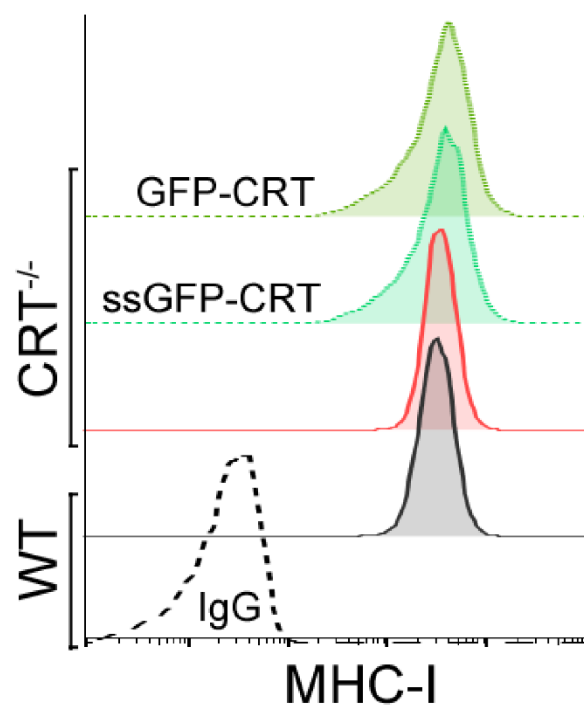

Supplement: Supplementary S2 [file cddis2016176x3.pdf]

Supplementary S3

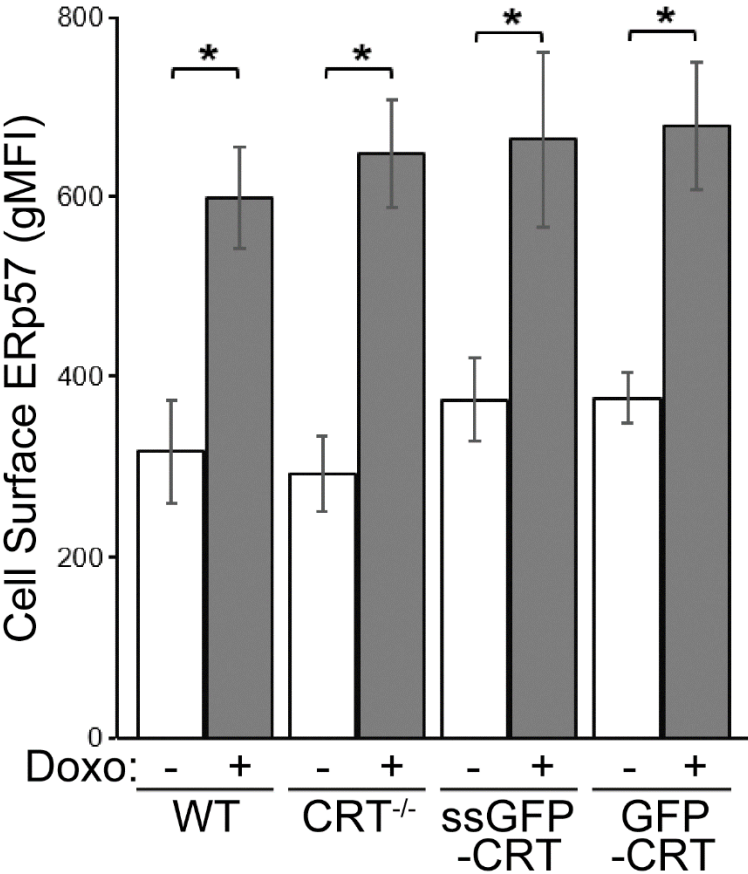

Supplement: Supplementary S3 [file cddis2016176x4.pdf]

## Supplementary S4

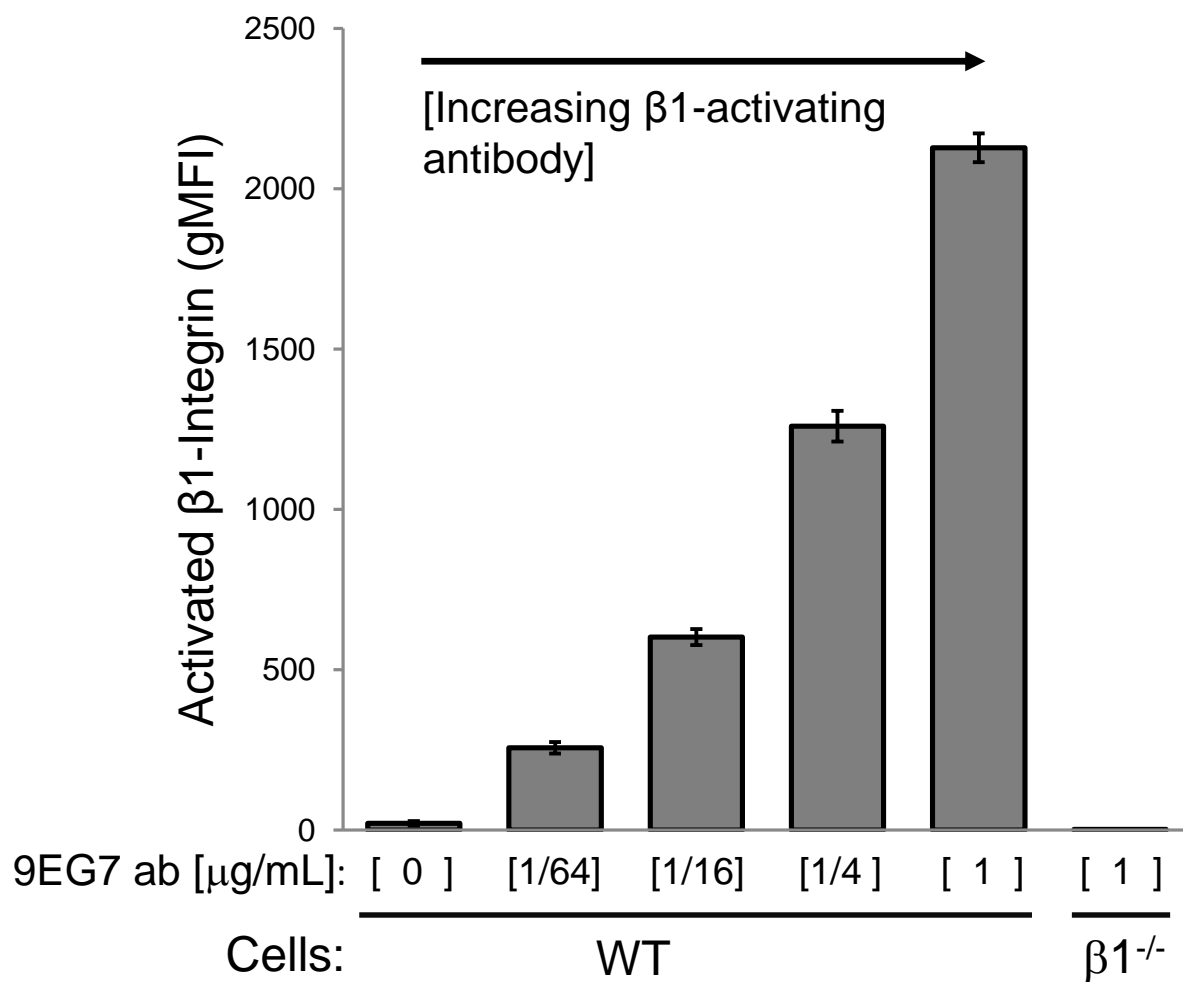

Supplement: Supplementary S4 [file cddis2016176x5.pdf]

Supplementary S5

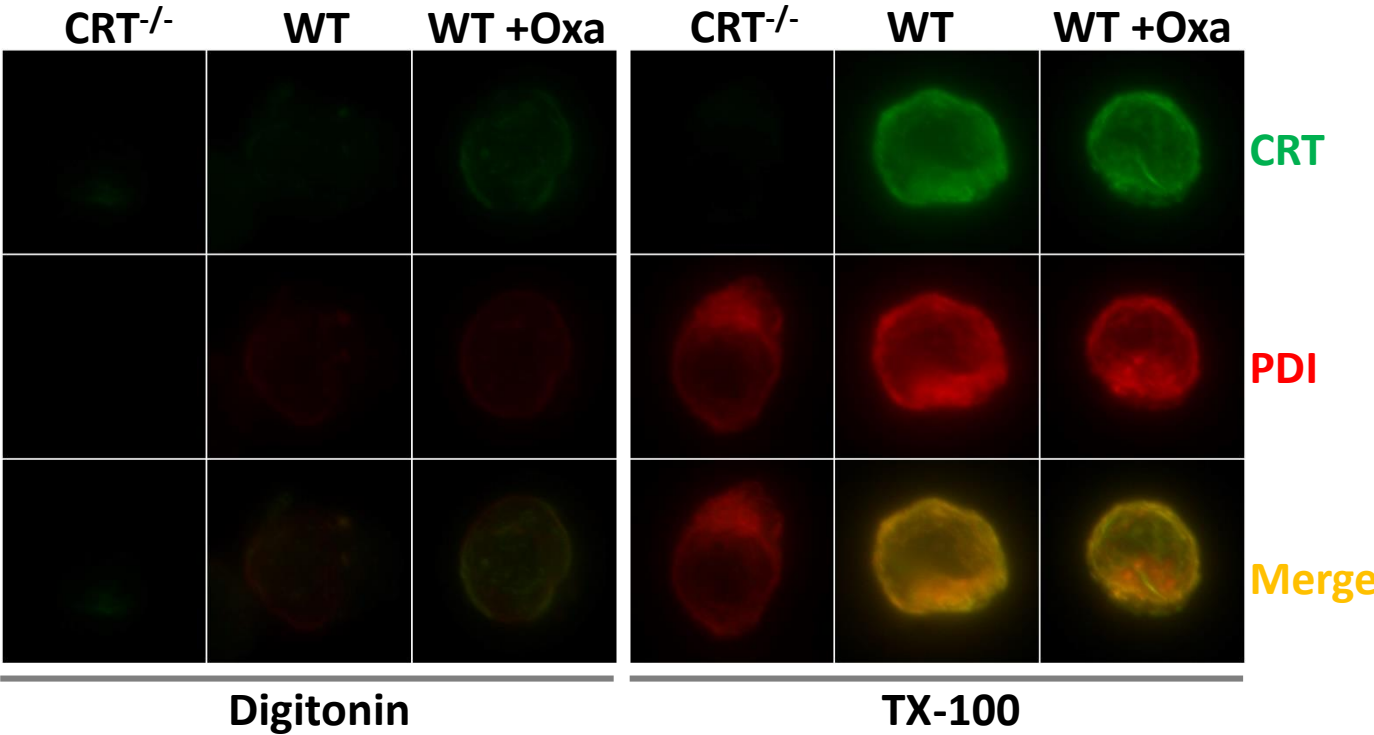

Supplement: Supplementary S5 [file cddis2016176x6.pdf]
